# “We do what we can do to save a woman” health workers’ perceptions of health facility readiness for management of postpartum haemorrhage

**DOI:** 10.1080/16549716.2019.1707403

**Published:** 2020-01-13

**Authors:** Fadhlun Alwy Al-beity, Andrea B. Pembe, Hilda A. Kwezi, Siriel N. Massawe, Claudia Hanson, Ulrika Baker

**Affiliations:** a Department of Global Public Health, Health Systems and Policy, Karolinska Institutet, Stockholm, Sweden; b Department of Obstetrics and Gynaecology, Muhimbili University of Health and Allied Sciences, Dar es Salaam, Tanzania; c Department of Community Health Nursing, Muhimbili University of Health and Allied Sciences, Dar es Salaam, Tanzania; d Department of Disease Control, London School of Hygiene and Tropical Medicine, London, UK; e Department of Family Medicine, College of Medicine, University of Malawi, Blantyre, Malawi

**Keywords:** Health facility readiness, postpartum haemorrhage, health worker perceptions, work-dynamics, quality of care, enabling environment, working environment

## Abstract

**Background**: In many low-resource settings, in-service training is a common strategy to improve the performance of health workers and ultimately reduce the persistent burden of maternal mortality and morbidities. An evaluation of the Helping Mothers Survive Bleeding After Birth (HMS BAB) training as a single-component intervention in Tanzania found some positive albeit limited effect on clinical management and reduction of postpartum haemorrhage (PPH).

**Aim**: In order to better understand these findings, and particularly the contribution of contextual factors on the observed effects, we explored health workers’ perceptions of their health facilities’ readiness to provide PPH care.

**Methods**: We conducted 7 focus group discussions (FGDs) and 12 in-depth interviews (IDIs) in purposively selected intervention districts in the HMS BAB trial. FGDs and IDIs were audio-recorded, transcribed and translated verbatim. Thematic analysis, using both inductive and deductive approaches, was applied with the help of MAXQDA software.

**Results**: Health workers perceive that their facilities have a low readiness to provide PPH care, leading to stressful situations and suboptimal clinical management. They describe inconsistencies in essential supplies, fluctuating availability of blood for transfusion, and ineffective referral system. In addition, there are challenges in collaboration, communication and leadership support, which is perceived to prevent effective management of cases within the facility as well as in referral situations. Health workers strive to provide life-saving care to women with PPH despite the perceived challenges. In some health facilities, health workers perceive supportive clinical leadership as motivating in providing good care.

**Conclusion**: The potential positive effects of single-component interventions such as HMS BAB training on clinical outcome may be constraint by poor health facility readiness, including communication, leadership and referral processes that need to be addressed.

## Background

Postpartum haemorrhage (PPH) is among the most common complications of childbirth and occurs in 6% of women [1]. It contributes to 20–50% of severe maternal morbidities worldwide and a quarter of all maternal deaths [2–5]. The biggest burden of PPH is in low-resource settings, including in Sub-Saharan African countries where more than 48% of PPH-related deaths occur [2,6].

Postpartum haemorrhage is an emergency where appropriate and timely management is crucial to prevent severe complications and death. Against the background of large increases in facility deliveries in many low-resource settings [7], health facility readiness to enable appropriate management of PPH is crucial [7]. The World Health Organization (WHO) defines health facility readiness as the availability of trained health workers, guidelines, equipment, essential medicines and supplies [8]. Health facility surveys have reported on poor readiness to provide PPH care according to WHO standards [9–12]. Several studies have also reported that health workers perceive poor health facility readiness as contributing to poor quality of care when managing life-threatening complications, including PPH [11,13].

Tanzania has one of the highest maternal mortality rates in the world with 398 [uncertainty interval 281–570] maternal deaths per 100,000 livebirths. A quarter of these deaths are due to obstetric haemorrhage, the majority caused by PPH [14–16]. The PPH case fatality is estimated to be high at 2.5% (range between 1% and 5.8%) in Tanzania [17], reflecting both poor access to care and sub-standard care once women reach the health facilities.

Tanzania has two clinicians and four nurse-midwives per 10,000 population and only 43% of the required health worker positions are filled [18,19]. Consequently, most of maternity and delivery units in the country do not have enough health workers to meet recommended numbers for delivery wards [19,20]. Lack of or inadequate skills have been commonly reported, both from poor pre-service training and from few, uncoordinated in-service trainings conducted in the country [21–24].

In-service training is an important strategy to improve health workers’ skills and clinical management of childbirth. In our recent publication [25,26], we reported improved practices and a reduction of severe PPH morbidities from 81% to 68% in intervention clusters following the implementation of Helping Mothers Survive Bleeding After Birth (HMS BAB). We used the HMS BAB basic curriculum that specifically addresses only prevention, early detection and basic management of PPH that was launched in 2013 [27]. Details of the training and trial are presented in Box 1.

While the improved practices and reduction of severe PPH morbidities are encouraging, it is important to gain a deeper understanding of the contextual challenges that may limit the potential effects of a single-component intervention such as HMS BAB. Several recent studies from Tanzania report on challenges in health facilities’ readiness to provide childbirth care, such as lack of a consistent supply of medicines, equipment and human resources [11,12,28–31]. In this context, it may therefore be reasonable to question the adequacy of single-component training interventions to address local challenges to provide quality PPH care. We therefore explored health workers’ perceptions of the readiness of their health facilities to provide PPH care, in order to better understand the specific context in which the HMS BAB was implemented and its potential contribution to the limited observed effects.

**Box 1.** The Summary of HMS BAB training and the HMS BAB Trial which was done in Tanzania [25–27]

**Helping Mothers Survive Bleeding After Birth (HMS BAB) in Tanzania** A competency-based 1-day training and uses a low fidelity simulator, Mama Natalie. The HMS BAB basic curriculum was used. This includes basic delivery care and AMTSL, assessment and basic management of excessive bleeding during childbirth but not the curative elements of Helping Mothers Survive Bleeding After Birth Complete (HMS BABC). In addition, health workers are trained to recognize women who need advanced care and referrals and to have a referral plan in place. Between 2014 and 2017, a cluster randomized trial evaluating the effect of the HMS BAB on PPH-related outcomes in Tanzania reported that when compared to the comparison clusters, intervention clusters had Significant and sustained reduction of proportion of women who suffered severe PPH morbidities from 81.8% to 68.3%, there was no change in comparison districts (70–71%). A higher proportion of women with severe PPH morbidities received intravenous oxytocin. Reduced long-term PPH case fatality during the postintervention period. The overall PPH case fatality remained high at 3.4%.

## Method

### Study design

We conducted a qualitative study based on 7 focus group discussions (FGD) with health workers and 12 in-depth interviews (IDI) with health-managers. We followed the COREQ-guidelines for reporting qualitative research [32].

### Study setting

Tanzania’s health-care system is decentralized to the district level, with district hospitals as the first referral level, supporting health centres and dispensaries. Over 70% of health facilities are publicly owned and managed under the President’s Office, Regional Administration and Local Government. The recruitment and deployment process, where district authorities identify local human resource needs, but the central government advertises, recruits and deploys health workers, is challenging [33]. Various cadres work in the maternity departments including clinicians (medical doctors, assistant medical officers, clinical officers) and nurses (assistant nursing officer – diploma, nursing officer – degree and nurse midwives).

All government facilities are mandated to procure medical supplies, laboratory reagents, and drugs from the Medical Stores Department (MSD) on a quarterly basis. Procurement from private vendors is allowed when MSD does not have the required supplies. There was a national blood transfusion services centre established in 2004 that coordinates blood transfusion activities through six zonal blood transfusion centres: Lake, Southern highlands, Southern, Central, Eastern and North-eastern [34]. In 2014, some of the formerly centralised activities such as community mobilisation and blood collection, were shifted to district authorities to reduce costs. The zonal offices continue to screen donated blood and perform quality control.

This study was done in the setting of HMS BAB main trial in 20 districts of Southern and Lake zones in Tanzania [25,26]. In 2016, 12 district trainers were trained for 5 days by Jhpiego master trainers well familiar with the typical facility and care provision structure in Tanzania. The one-day in-facility training was done in pairs, each pair was supported by Jhpiego master trainers during the first two trainings. All health facilities used same training schedule but adapted to the facility. Training technique emphasized practices and brief knowledge sessions. Each skill training was followed by feedback sessions. In each facility, two health workers were selected and oriented to be the local peer practice coordinators during an additional half-day session. They were trained on using the Mama Natalie kit and the training materials for weekly practices. The peer practice coordinators organized and led weekly practices in their health facilities for 6–8 weeks. During each session’s health workers discussed and received feedback on their simulated skill performance.

To give an overview of the study context, Table 1 displays the availability of different health facility readiness components in the HMS BAB trial sites: 20 districts in 4 regions of Tanzania, Mtwara, Lindi (Southern zone) and Mwanza, Simiyu (Lake zone) with a total of 61 health facilities. Oxytocin, the main drug for PPH management, was largely available, while medical supplies such as gloves, blood test reagents, and basic equipment were missing in most health facilities.

**Table 1.** Availability of resources necessary for management of Postpartum haemorrhage

	Health Centre	Hospital	All
	*n* = 38	*n* = 23	*n* = 61
	*n* (%)	*n* (%)	*n* (%)
Public ownership	35 (92)	16 (70)	51 (84)
Supervision by district management in the last 6 months	36 (95)	NA	NA
**Availability of physical infrastructure**			
Operation theatre operating on 24/7 basis	10 (26)	22 (96)	32 (52)
24 hours operating laboratory	37 (97)	23 (100)	60 (98)
24 hours light source in the labour ward	16 (42)	15 (68)	31 (52)
Available motorized transport stationed at the facility	13 (34)	19 (82)	32 (52)
Facility communication used in last referral	8 (21)	6 (26)	14 (23)
**Availability of blood transfusion services**			
Haemoglobin level testing	35 (92)	23 (100)	58 (95)
Blood grouping reagents	11 (45)	17 (94)	28 (67)
HIV and Syphilis testing	23 (61)	21 (91)	44 (72)
Blood for transfusion	10 (26)	23 (100)	33 (54)
**Availability of drugs and medical supplies**			
Oxytocin (first-line uterotonic drug)	32 (84)	23 (100)	55 (90)
Misoprostol (second-line uterotonic drug)	7 (18)	12 (52)	19 (31)
Intravenous fluids for resuscitation	31 (82)	22 (96)	53 (87)
Long armed gloves (for manual placenta removal)	15 (40)	15 (65)	30 (50)
Sterile gloves	24 (63)	19 (82)	43 (71)
**Availability of equipment**			
Suture trays, speculum and light for assessing perineal and cervical tear and repair	6 (16)	8 (35)	14 (22)
BP machines	29 (76)	22 (96)	51 (84)
**Availability of guidelines and protocols**			
Protocol for PPH management (with appropriate dose, administration route and alternative/second line drug)	15 (41)	17 (77)	54 (32)
Written referral protocols	21(8)	10 (45)	30 (18)

Blood transfusion services were available in all hospitals and 26% of health centres. On average, health centres had 7 units [IQR 3–19, n = 10] of blood on the day of assessment and hospitals had 10 units [IQR 5–19, n = 23]. Standard PPH management protocols were available in 41% of health centres and 77% of hospitals. Only one-third of health facilities had a written referral protocol.

Staffing levels were below national recommendations in nearly all health facilities, which was more pronounced in health centres (Table 2).

**Table 2.** Facility staffing and delivery loads

Availability of human resources anddelivery case loads	Health centres *n* = 38 [IQR]	Hospitals *n* = 23
Median number of clinicians^a^ per facility	1 [0–1]	3 [1–6]
Median number of nurses^a^ per facility	2 [1–3]	8 [5–9]
Median total deliveries per month per facility	37 [19–79]	226 [139–287]
Median normal deliveries per month per facility	37 [19–79]	182 [103–253]
Median load (deliveries per nurse, per month)	17 [10–26]	22 [15–35]
Mean load	22 ± 18	30 ± 22

^a^Nurses include nurse-midwives, assistant nursing officers and certified nurses. Clinicians include medical doctors, assistant medical officers and clinical officers.

For this study, we purposefully selected 4 out of the 10 intervention districts included in the HMS BAB trial. We balanced respondents from the two zones, urban and rural districts, and facility readiness aspects. Initially, we aimed to select districts according to extremes in availability of drugs and supplies to manage PPH, but the low variability between districts made such theoretical sampling impossible.

### Study participants

For FGDs, we selected participants from the same facility level, such homogeneity maximised the potential for an open discussion about day to day experiences specific to the level of facility. We planned to have 2 FGD per district, one at health centre level and another at hospital level. Seven out of the planned eight FGDs were conducted. In one district we could not get health workers to sit together as they were all absent for long periods. Each FGD comprised 5–7 health workers involved in the provision of maternal health care. The IDIs with health managers included regional and district medical officers and reproductive, maternal, newborn and child health coordinators.

All FGD participants were purposefully selected from the labour wards and were recruited through the facility administrators. They were contacted a day in advance and requested to meet at the facility at a given time. Due to shortages and long working hours for health workers, it was agreed that FGDs would be conducted during working hours with one health workers remaining on the ward and others participating in the discussions. To maximise participant numbers, FGDs were scheduled during the shift change to enable participation of both incoming and outgoing staff. Depending on the availability of health workers, FGDs included a mix of cadres; five FGDs had clinicians and nurses or nurses and medical attendants and two FGDs had nurses only (Table 3).

**Table 3.** Participant characteristics

	Focus group discussions	In-depth interviews
Number of participants	51 (in 7 focus group discussions)	12
Age (median years)	Median 33 years, (range 23–57 years)	Median age 41 years (range 27–54 years)
Gender (female/male)	45 Female (88%), 6 male (12%)	6 Female (46%), 7 male (54%)
Years of work experience in maternity ward	Median 3 years, (range 0–30 years)	Median 8 years (range 1–18 years)
Cadres	5 Clinicians, 32 nurse/midwives,13 medical attendants, 1 Maternal Child Health Aid (MCHA)	6 Clinicians, 7 nurse-midwives

### Data collection process

All data collection was conducted between March and May 2017. All participants were informed of the study purpose, measures put in place to respect confidentiality and their right to withdraw at any time. An information sheet about the study was provided and read out by one of the interviewers. All respondents chose to participate and signed written informed consent. They also consented to the audio recording of the interviews and FGDs.

Topic guides (Appendix A for FGD and Appendix B for IDIs) prepared by FA with input from UB and CH and translated into Swahili by FA were used. The first author (FA) and two research assistants conducted the FGDs and IDIs in Swahili. The two research assistants, male and female, are social scientists with experience from several qualitative studies in Tanzania and were not part of the HMS BAB trial team. Interviews were held in a private area within the health facility with interviewer and participants only, were audio recorded, and supplemental notes were taken during the discussions. On average, the FGDs lasted 55 min, and the interviews 40 min. The recorded interviews were transcribed and translated verbatim by the research assistants. FA performed quality checks in over half of the translated transcripts to ensure preservation of the original meaning. Transcripts were not shared with respondents.

The number of interviews was pre-determined; however, it was felt that saturation in the interview material was reached before the last few interviews were conducted as no or little new information emerged. The interviews were listened to on a daily basis. No repeat interviews with respondents were done.

### Analysis

Transcripts were analysed by the first author (FA) with frequent and substantial input by the senior author (UB). MAXQDA 12 software was used to aid the analysis. Transcripts were initially read and re-read to capture the whole, then coded inductively with labels closely derived from the transcripts to preserve their meaning [35]. Similar codes were then grouped into categories and sub-categories. Throughout the process, transcripts were re-read to clarify emerging codes and to triangulate the information between FGDs and IDIs. Categories were finally grouped under two themes which were created partly inductively but partly deductively having the definition of health facility readiness in mind [36].

A substantial proportion of transcripts was independently read and coded by the two investigators FA and UB. Codes, categories and themes were then discussed and harmonised which included re-coding of some transcripts.

## Results

Two major themes emerged during the analysis: (1) Inconsistent availability of resources limiting provision of care and (2) Management of women with PPH is prioritized. Theme 1 had four categories with three to four sub-categories, reflecting availability of drugs and supplies and blood as well as health workers and functionality of referral systems (Table 4). Theme 2 had three categories each with two to three sub-categories reflecting on organizational and working process around PPH care.

**Table 4.** Themes, categories and subcategories

Theme	Category	Sub-category
I. Inconsistent availability of resources limiting provision of care	Drugs and supplies for PPH management not always available	Stock outs in supplies Drug availability varies Delays in procurement
	Availability of blood fluctuates	Not enough blood collected Delayed processing of blood Challenges in specific blood groups
	Unclear and unreliable referral system	Unclear referral pathways No communication before referral Inconsistent availability of transport Health workers argue during referrals
	Few and unsupported health workers	Not enough health workers Work in maternity is overwhelming Perception of health workers’ poor skills and attitudes Perceived insufficient support from supervisors
II. Management of women with PPH is prioritised	Women with PPH are prioritised	Understanding PPH is an emergency Working as a team Supporting each other
	Supportive leadership is helpful	Good leadership Supervisors improvising local solutions

### Theme 1: inconsistent availability of resources limiting provision of care

Fluctuations and inconsistencies in availability of resources: medical supplies, drugs and blood result in poor health facility readiness to manage PPH. Health workers perceive that such inconsistencies negatively affect the provision of care for women suffering from PPH.

#### *Drugs and supplies for PPH management not* always *available*

Health workers describe an environment where fluctuations and shortages of drugs and supplies are common. This variability is perceived to be a result of poor internal allocation and inefficient procurement processes due to poor communication and lack of technical capacity within facilities.

When the HMS BAB trainers came and trained us we learned to do assessment to identify excessive bleeding and try to identify the cause of that PPH … is it a tear or atony, you need gloves to assess … you may find that you need to ask relatives to go bring gloves, during that time the woman continues to bleed … [FGD 6]

Health workers experience frustrations when they have to ask relatives to buy supplies from outside shops, especially at night.

Health managers also express their frustration with administrative barriers in their dealings with MSD. Frequently, an ‘out of stock’ response is given for quarterly orders and there is a long bureaucratic process to procure from private vendors.

#### Availability of blood fluctuates

##### Not enough blood collected

Both health workers and managers describe that blood transfusion services have improved compared to previous years. Some health centres have been upgraded to provide blood transfusion and districts or health centres with excess blood units redistribute these to facilities with a higher need. However, major issues were described in recruiting donors. Lack of awareness in the community, poor knowledge and misconceptions about HIV screening however deter people from donating.

I remember a woman with blood group O negative […]. She said her relatives refused to donate because of fear to be tested for HIV while they don’t want to. This is still a problem for the people of this zone. [FGD 7]

Due to blood supply shortages, health workers require relatives to ‘replace’ blood, if their patient had received blood transfusion as part of PPH management, but this requirement usually remains unfulfilled. Sometimes relatives are required to donate blood before their patient is admitted for elective surgery to avoid shortage.

##### Delayed processing of blood samples

Samples from collected blood units are sent to zonal blood transfusion centres for screening (syphilis, HIV and Hepatitis B and C). Blood should be used within 1 month of donation, but health workers report that the screening process often takes long and therefore reduces the period available for blood to be used. This disconnect between the zonal blood transfusion centres and the district blood teams are perceived as a root cause for the lack of blood for transfusion.

But in the council, there are challenges, like council (name removed) collected blood on Monday but sent samples to be screened on Thursday until they were out of stock. They don’t bring samples in time. [IDI 4]

##### Challenges in specific blood groups

Challenges exist with availability of specific blood groups or blood products, limiting the management of women with PPH. Laboratory technicians keep a registry of people with rare blood groups who can be contacted during emergencies. Some donors require to be paid for donating and sometimes relatives do pay such donors.

The laboratory technician knew a man who was blood group O negative. He (donor) wanted to be paid and the woman’s husband agreed to pay. She was later referred to [name of health facility omitted] for more transfusion. [FGD 5]

#### Unclear and unreliable referral system

A functioning referral system requires communication within and between facilities as well as means of transport from one facility to another.

##### Unclear referral pathways

Health workers in health centres express that they often need to refer cases that require advanced care due to poor resources and lack of skills, and because of feeling overburdened by mothers being referred to them from dispensaries. The referral chain within the district is not perceived to be clear. For example, women will go to health centres rather than dispensaries so that they can get transport to a higher level if required.

##### Inconsistent availability of transport

Arranging transport is not easy and barriers such as lack of fuel, driver being off-duty or absent for other reasons, broken ambulances or the ambulance being busy, are often encountered. At times, health workers ask relatives to contribute to pay for fuel.

When there are no funds for fuel and the ambulance is available, relatives contribute money for fuel about 80,000/= shillings (35USD). [FGD 1]

##### No communication before referral

The communication between facilities during referral is experienced as difficult. A telephone landline is usually not available, health workers themselves may not have call credit on their mobile phones, or experience network problems, especially in rural areas. When health workers manage to call, they may not have the right number or a direct number to a health worker who may not be on duty, making communication for referral challenging.

We presented the communication challenges during a meeting. They (supervisors) decided to give us phone number of the supervisor (of referral facilities). We wrote the number on noticeboard, unfortunately these numbers were reachable on the first days only, after that they weren’t reachable. I personally took a doctor’s contact, when you call, he tells you he is not on duty and that just bring the patient if it is an extreme emergency. [FGD4]

##### Health workers argue during referral

Health workers at the receiving facilities are frustrated by the communication challenge during referral. They rush to save the referred mother while needing to continue to provide care to patients already in the facility. Consequently, a hostile confrontational relationship between health workers from the two levels occurs. Accompanying health workers feel harassed for not ‘doing their job’, sometimes forced to stay and continue working at the referral facility. Additionally, feedback mechanism between these facilities frequently takes the form of reprimand rather than to foster collaboration.

They (health workers at receiving facilities) think we are avoiding our duties to help a patient by referring. By that time, you have struggled and concluded that the woman is going to die on your hands, she needs more advanced care. But those [health workers at the receiving facility] receiving think differently. [FGD 4]

#### Health workers are few and unsupported

##### Not enough health workers

Poor availability of skilled health workers is perceived to be a challenge by all. Health managers feel frustrated that fewer health workers than requested are posted to the facilities, and typically health workers of lower cadres than needed. Furthermore, better skilled health workers often request transfers to urban and semi-urban areas; few agree to work in rural areas due to its non-conducive environment.

In many rural facilities, clinical or medical attendants, a cadre with a one-year-training only, are therefore found providing the bulk of services although the government aims to phase this cadre out.

A challenge for us medical attendants, we are not qualified to attend to a pregnant woman. But we are the ones giving service to these women [laughter]. Today’s matron for example, is a medical assistant, of course she is just acting … the real matron has gone to the circumcision seminar … [FGD 6]

##### Work in maternity is overwhelming

Most health workers perceive work in the maternity ward as overwhelming and intense, especially when there are few health workers working long shifts. Exhausted and overwhelmed staff may compromise care as patient monitoring is not a priority resulting in late detection of complications.

Sometimes, you find that a provider has doubled (her shifts), working from morning till night. Once her mind is tired, she can do mistakes, she will give poor quality service, because her thinking capacity is exhausted.… She is overwhelmed by a lot of work. [IDI 5]

##### Perception of health workers’ poor skills and attitudes

Some health managers perceive that health workers perform poorly and complain unnecessarily. Their view is that the maternity wards are sufficiently staffed by health workers but health managers with a clinical background were more empathic as also described by the quote above.

##### Perceived insufficient support from supervisors

Overall, health workers describe being dissatisfied with the working conditions and feel unappreciated despite working hard. Some of the health managers prefer administrative work and refuse to do clinical work, even when there are is health workers shortage.

She (supervisor) does not work. If you are on same shift as her you consider that you are alone. That is a liability. Everywhere they know she does not work […]. One day we were busy and she was requested to come and help delivering the babies. She refused to work because of her rank. Later, she came to ask, ‘how many fresh stillbirths did you have today?’ [FGD 7]

Health workers feel that they are blamed and threatened when a bad outcome happens. The threats come from supervisors and managers as well as politicians, and repercussions range from being demoted to being transferred to a more rural facility or being subjected to legal action.

When a woman dies in a health facility, we go look at availability of medical supplies, health-care providers, infrastructures, guidelines and then attitudes. Sometimes the problem is the health provider. We have taken legal action at some places … [IDI 3]

### Theme 2: management of women with PPH is prioritized

#### Women with PPH are prioritized

##### Understanding PPH is an emergency

Many health workers express an understanding that PPH is a life-threatening condition and requires teamwork. They describe shouting for help and starting resuscitation while calling for a senior colleague to assist. Sometimes, health workers use personal resources such as mobile phones to call for additional staff.

When they heard ‘call for help’, people were organized, no one was unsure of what to do or ignored the call. There was a problem. People stood up, came to the problem and worked together until the woman was safely referred to higher level. That mother survived. [FGD 7]

##### Working as a team

In some cases, health workers prolong their shifts to support their colleagues during PPH emergencies, staying until management or referral is complete. No overtime payments are expected during such instances and health workers feel professionally bound to see these women survive. There is also an experience of good support from laboratory technicians, who will sometimes work beyond their normal hours to ensure that blood for transfusion is made available.

We work as a team. The last PPH we had was very severe. Although my shift was over, I stayed to help until the patient was stable for referral and I accompanied her. [FGD 4]

##### Supporting each other

Respondents describe that when PPH happens they usually support each other and strive to save a woman. They know most PPH cases cannot be predicted.

#### Supportive leadership

##### Good leadership

Some supervisors work hand in hand with their health workers regardless of their position and help in routine as well as emergency situations. Some health workers describe leaders who are always ready to give advice and assist, even when they are off-duty. Such leaders are perceived to inspire good performance and teamwork in day-to-day activities.

Some supervisors motivate you, like our matron she does all the duties and does not ask you anything or argue with you. You feel obliged to work alongside her. She leads by example and really works. [FGD 5]

Health managers described that many health workers do not have adequate skills. Even after different in-service trainings, some of which use classroom based-training, health workers need support to maintain what they have learned and improve. There is a need for continuous clinical mentoring and coaching.

The issue of coaching and mentorship … health workers need to be reminded regularly. They need to have things in their fingertips such that when there is a PPH, they know what to do and at what order. [IDI 2]

##### Supervisors improvising local solutions

Some health managers at the district level find local solutions for day to day challenges; for example, ‘transport-officers’ have been appointed in some districts to organize transportation during an emergency referral. Health managers manage drug shortages in specific health facilities by relocating the available stock from health facilities that have excess to those with stock-outs. Such arrangements work when there is an acute shortage.

If there is oxytocin stock-out … then we see how to do re-allocation between facilities. We communicate with each other and frequently with the region. [IDI 5]

## Discussion

### Overall findings

We explored health workers’ perceptions of health facility readiness to better understand the observed effects of the HMS BAB single-component intervention. Health providers described low readiness in all components at the facility level such as shortages of medical supplies, equipment and drugs, poor availability of blood for transfusion, and poor referral infrastructure such as communication and transport when referral for advanced care is required. Such challenges led to suboptimal management of women with PPH despite health workers having had the training. In addition, challenges in collaboration, communication and leadership support were raised, complicating management of cases within the facility as well as in referral situations leading to perceived stressful working conditions. In some health facilities, health workers perceived supportive clinical leadership as motivating in providing life-saving care to women with PPH.

#### Medical supplies, drugs, equipment and blood

Several studies from LMIC, similar to ours, highlight that the unavailability of medical supplies, drugs and equipment constrain timely and responsive care and also demoralise and disempower health workers [11,12,24,30,37,38]. Bailey et al. associated low facility readiness with high rates of referral of all difficult cases [39].

Death from PPH can occur within few hours after the onset. Interventions for fluid replacement, arresting the cause of bleeding and in severe cases, rapid blood transfusion to replace lost blood can be life-saving. Over a quarter of maternal deaths from PPH have been reported to be contributed to lack of blood [9,40]. Low availability of blood in health facilities as seen in this study prevents timely care and contributes to poor outcomes. Health workers raised concerns that the low availability of blood transfusion is often caused by dysfunctional logistics. Similar findings of low blood availability have been reported in the country [29,40]. The need for frequent blood transfusion could potentially be reduced if skilled health workers are able to prevent the incidence of severe PPH and do basic management as demonstrated by HMS BAB training [40,41].

#### Referral system

While health workers expressed their struggle with shortages of supplies, more often they raised the issue of malfunctioning referral processes that constrain the provision of care. We found many gaps in the existing referral system including lack of functional transportation and communication processes, unavailable referral protocols, lack of collaboration between referral levels and lack of accountability and supervision when referrals were concerned. These challenges were also described in a literature review of maternity referral systems in developing countries by Murray et al. [42]. In our study, lengthy referral and unclear referral processes were perceived as a barrier to appropriate care for women with PPH. Health workers expressed distress over negotiating the referral process with several challenges: a) unavailability of ambulances, b) drivers or fuels, c) long waiting time and d) distance travel. Similar challenges have been highlighted in other studies [17,37,43,44].

#### Unsupported, unmotivated and unskilled health workers

Similar to other studies in LMIC, this study revealed perceptions of an insufficient number of skilled health workers who are overworked, poorly paid and demotivated [37,45,46]. Both health workers and some health managers described that with many patients and few staff, some clinical duties which do not require immediate attention are neglected. For example, routine monitoring of postpartum women is not a priority; at times women develop PPH and are incidentally found late.

The frustrations created from poor supervision, through limited support in solving day-to-day challenges, a focus on fault-finding, blame and reprimand, have been reported in several other studies from LMIC [37,46,47]. Clinical mentorship and supportive supervision, on the other hand, has been associated with improvements in health worker performance and satisfaction as it focuses on every day clinical practice and is responsive to health workers’ needs in everyday practice [48–50].

Our findings point to a need to explore the dynamics and relationships between health managers and health workers, to identify ways to improve the working environment. Some health managers perceived health workers’ lack of skills and poor attitudes to be the underlying cause of poor practices rather than lack of resources. Other studies from Tanzania and from other LMIC have also reported a lack of collaboration between different cadres working in the delivery wards [37,47,51]. The resulting distrust and practice of blaming and reprimand need to be addressed. Studies investigating management strategies that focus on support and communication aspects have shown to improve work relationships and health workers’ perceptions of improved performance [49,52].

In several instances, health workers appreciated being able to work together when faced with high-risk situations such as PPH. We think this teamwork could be associated with the HMS BAB training that study facilities received as an intervention. Several studies report better patient outcomes when health workers of different cadres’ train and work as a team [53,54].

While in-service trainings are credited to improve health workers' skills in managing PPH, such skills cannot be fully utilized and implemented when health workers are struggling with organizational and work processes within their health facilities. Training interventions, unfortunately, often neglect these management and organizational aspects as they concentrate on clinical skills and competences.

### Methodological considerations

The purposive sampling of respondents, including health managers and health workers of different cadres from different geographical zones and different levels of health facilities, ensured variability in our data. The use of FGDs and IDIs yielded rich descriptions from variety of respondents. All authors had some experience of working in the Southern zone, while FA, ABP, and HK had some experience of working in the Lake zone.

One of the researchers (FA), obstetrician/gynaecologist working in a university hospital in Tanzania, had been collaborating with respondents’ health facilities throughout the two-year HMS BAB trial, facilitating the contact but also introducing a risk of social desirability bias.

The process of coding, re-cording and grouping into sub-categories and categories was iterative and involved input from three of the authors increasing analytical rigor.

We also had the opportunity to triangulate perceptions of health facility readiness with the quantitative findings from the HMS BAB health facility surveys as described in the study setting. While the IDIs and FGDs were done at the end of the HMS BAB trial, we also had data from two health facility assessments during the HMS BAB trial period and there was no evidence of change in health facility readiness during this time, that may affect the accounts shared by respondents [25].

An important limitation was that we did not include mothers’ or families’ perspectives or those of supportive staff outside of the delivery ward.

## Conclusion

Our study has highlighted the challenges of health facility readiness beyond the commonly described shortages of material and human resources. It illustrates the crucial role of clear and effective referral systems that includes respectful and supportive communication between health workers. Incorporating components to improve team dynamics, relationships and collaboration into in-service training focusing on clinical skills may therefore be a feasible approach to support health workers to translate their newly acquired skills into practice, even where the lack of medicines and supplies remains a challenge.

## Paper context

The HMS BAB trial in Tanzania reported a modest reduction in PPH severe morbidities and mortality. We explored health workers’ perceptions of the health facilities’ readiness to provide PPH care. Health workers perceived low readiness which hinder provision of care despite having acquire skills during training. Future training interventions on PPH care should integrate additional aspects of health facility readiness to address team dynamic, working relationships and collaborations to have a meaningful and sustained effect.

## Appendix A. Focus Group Discussion topic guide for Health workers.

*We know that PPH can happen at any time, requiring prompt action from health-providers, supplies and medication and resource mobilizations. I will like you to remember the last case of postpartum haemorrhage managed in your facility*.

1. Could you share your experience in managing this case?

***Probe:***

*What was good and made you happy with the way you managed the case? What were the challenges?*

2. Can we talk about medical supplies and drugs needed for PPH What is your experience in getting them when needed?

***Probe***:

*Are these supplies always available? What happens at night or during public holidays? How will you describe use of laboratory and their response during management of PPH case?*

3. What has been the experiences when you have needed blood and blood products in an emergency situation like PPH.
4. Perhaps most of us have at one time referred a woman with postpartum haemorrhage to a higher-level facility.

***Probe***:

What are the issues on this, what are the challenges? Who authorizes transport, how do you feel about this, how do you deal with the challenges?

*Would you say you are happy with the current referral system? Why? How can this be improved?*

5. Can you talk a bit about the way you interact with facility administrators?
6. We learned that rotation of staff is at times a problem. What has been your experience on staff rotation?

## Appendix B. In-depth Interview topic guide.

1. In your opinion, why is postpartum haemorrhage still a problem in the area?
2. We stress that we need providers with skills and knowledge for emergency obstetric conditions including PPH. To this the ministry and stakeholders have and are training a lot. In your opinion, have these trainings helped in terms of better care? Why? How? Do you have a record of those trained? How are they accountable?
3. We saw that there is staff rotation between different sections, what has been your experience as an administrator?
4. Some providers have reported high burn out working in the labour ward and stress due to high load and high blame area, have you come across such issues? What have you done?
5. Can we discuss the challenges of blood availability and use in your district?
6. I will also like to know a little bit more on the timely availability of drugs and medical supplies including oxytocin and misoprostol.
